# Superior Vena Cava Syndrome Due to Right Anterior Mediastinal Hematoma: A Case Report

**DOI:** 10.7759/cureus.26994

**Published:** 2022-07-18

**Authors:** Ramy Ibrahim, Swarada Yadav, Sumaita Waqar, Jose Ruben Hermann, Abeer Sarwar, Sundeep Shah

**Affiliations:** 1 Research, Premier Medical Associates, The Villages, USA; 2 Internal Medicine, University of Alabama at Birmingham, The Villages, USA; 3 Dow Medical College, Civil Hospital Karachi, Karachi, PAK; 4 Internal Medicine, Premier Medical Associates, Ocala, USA; 5 Internal Medicine, Fatima Memorial College of Medicine and Dentistry, Lahore, PAK; 6 Internal Medicine/Nephrology, Premier Medical Associates, The Villages, USA

**Keywords:** epicardiac wires of cabg, computed tomography, pacemaker leads, mediastinal hematoma, superior vena cava syndrome

## Abstract

The superior vena cava syndrome (SVCS) has been frequently reported to be secondary to malignancy, specifically, small cell bronchogenic carcinoma and non-Hodgkin's lymphoma. There is some data suggesting causes like postprocedural hematomas. We aim to describe a case of a patient who developed SVCS secondary to a mediastinal hematoma secondary to epicardial pacer leads (postprocedural).

Our case is about a 75-year-old male with a past medical history of coronary artery disease and coronary artery bypass graft (CABG) who presented to the Emergency Department (ED) with moderate-to-severe right axillary pain radiating to the ipsilateral side of the neck, arm, and chest, associated to right temporal headache. A computed tomography angiography (CTA) of the chest was indicated at the time and revealed a hematoma with an active extravasation within the right superior anterior mediastinum, outside the pericardium. The patient was admitted to the Cardiovascular Intensive Care Unit (CVICU) and was started on nicardipine as his blood pressure in the ED was 217/125 and remained elevated despite proper pain management. A repeat CT scan of the chest showed a regressing hematoma that coincided with an improvement of the symptoms.

This case highlights the importance of the complications of anterior mediastinal hematoma. The superior vena cava syndrome can develop after cardiologic procedures, after the implantation of devices. Prompt clinical diagnosis, including imaging, and treatment are necessary to manage this condition.

## Introduction

The superior vena cava (SVC) is formed from the union of the left and the right brachiocephalic veins and is responsible for draining deoxygenated blood from the chest, head, neck, face and arm to the right side of the heart [1]. The superior vena cava syndrome (SVCS) comprises signs due to the obstruction of the drainage of blood due to a clot or a space-occupying mass leading to extrinsic compression of the SVC [1]. The most common causes of the syndrome are carcinogenic, including small cell bronchogenic carcinoma, followed by Hodgkin's and non-Hodgkin's lymphoma [2]. Among the non-carcinogenic causes of SVC syndrome are thrombus formation due to pacemakers, implantable cardioverter-defibrillators (ICD), chemotherapy, hematomas or any agent leading to stasis of the blood or narrowing of the vein itself [3]. It is usually diagnosed clinically based on a wide range of central nervous system obstruction symptoms [3]. The constellation of symptoms can range from asymptomatic to presenting with swelling of the face, neck and arms due to lack of drainage from these parts along with difficulty breathing and hoarseness of voice [1,3]. These symptoms can be gradually progressing and are very often missed [2]. The superior vena cava syndrome is also known to cause sudden death [3,4].

## Case presentation

**Figure 1:**
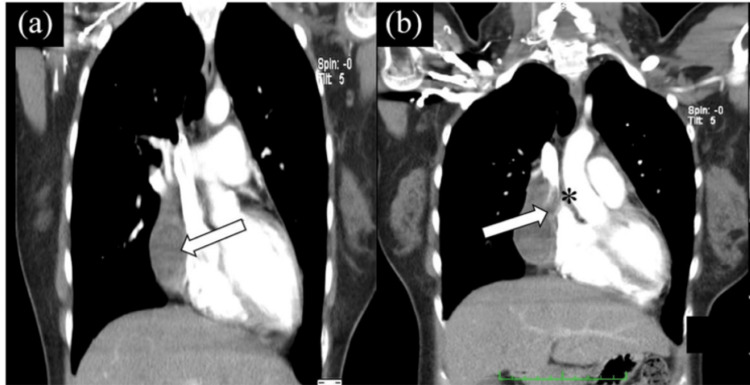
CT scan shows expanding anterior chest hematoma (arrow) compressing SVC (asterisk): a) expanding hematoma compressing SVC; b) collateral vessels visualized Source: Saboe et al. [4] SVC: superior vena cava

A 75-year-old male with a past medical history of coronary artery disease status post coronary artery bypass graft (CABG) presented to the ED with right axillary and chest pain. The pain started as a right temporal headache that was radiating to the right side of the neck, the right shoulder and the right arm. The patient also complained of severe weakness in his right arm and inability to move the arm. The patient provided a history of lifting a heavy object prior to the onset of symptoms. Upon arrival in the ED, the patient was hypertensive and tachycardic. On further examination, the physical exam findings were unremarkable except for mildly decreased breath sounds in the right lung base with dullness to percussion. The neurological exam was intact. The pain subsided when the patient was given hydromorphone. A series of blood tests and imaging were done in order to find the cause of the pain and weakness on the right side. These tests included complete blood count, comprehensive metabolic panel, lipid panel, troponin levels, and urinalysis. The patient had a declining hemoglobin trend during his stay in the hospital. To rule out acute coronary syndrome, troponin levels were checked and were found to be negative. Electrocardiography (EKG) showed nonspecific ST changes. CT chest without contrast showed right pleural effusion with hemopericardium, which was confirmed with contrast. The computed tomography angiography (CTA) chest revealed a hematoma with an active extravasation within the right superior anterior mediastinum measuring 6 x 8 x 4 cm, outside the pericardium, and helped rule out hemopericardium. The imaging, just like in Figure 1, also showed that the posteriorly superior vena cava was compressed [4, 5]. Due to the presence of hemorrhage adjuvant to epicardial pacer leads, it was proposed as a possible etiology for hemorrhage.

CT brain showed no abnormality. An echocardiogram showed an ejection fraction of 60% and confirmed the absence of hemopericardium. The patient was admitted to the cardiovascular ICU (CVICU) and was started on nicardipine as the blood pressure in the ED was 217/125. Repeat CT showed regressing hematoma with an improvement of the symptoms.

With the progressive improvement in the condition of the patient and hemodynamic stability, he was discharged from the hospital and was recommended to follow up with the primary care physician for long-term management. The entire diagnosis, treatment and management of the patient required four days of hospital stay without any acute event.

## Discussion

Mediastinal hematoma caused due to damage from epicardial wires of CABG is an uncommon but significant complication [4, 5]. Mediastinal hematoma with sufficient volume may result in compression of the neighboring heart chambers or vessels, one of which could be superior vena cava leading to the superior vena cava syndrome [5]. 

The superior vena cava syndrome is an infrequent presentation and thus can be easily missed [1]. The most common sources of SVCS include clot formation that leads to obstruction of blood flow returning to the heart [6]. The increasing use of central catheters and pacemaker wires in the past few decades has led to an increase in the cases of clot formation [6,7]. The management of SVCS must be according to the underlying etiology. Management should aim towards the removal of the block in the vein and improving the drainage of the blood through the SVC [8]. Most of the patients may present life-threatening symptoms like difficulty swallowing, impaired cough, and shortness of breath, and require immediate intervention like endovenous recanalization with stent placement in order to improve the blood return to the heart and avoid death due to respiratory failure [9,10].

In this case, the epicardial pacer wires led to local anterior mediastinal hematoma which caused blockage of the SVC resulting in SVCS [5]. The presentation of this patient with chest pain radiating to the neck, shoulder and arms and ipsilateral right headache along with findings on CTA supported the diagnosis of SVCS [1, 5]. The goal was to avoid hypertension to prevent worsening of the hematoma and resultant SVC symptoms. Due to a decrease in the hematoma, as demonstrated on repeat imaging, no surgical intervention was needed [5].

## Conclusions

The superior vena cava syndrome should be considered in patients presenting with chest pain, swelling of unilateral side, weakness in the upper extremity and other superior vena cava blockage symptoms with a history of cardiac implant placements. This case emphasizes the significance of complications of anterior mediastinal hematoma caused due to cardiac devices. The expanding hematoma can compress the surrounding structures leading to life-threatening complications like superior vena cava syndrome. Prompt clinical diagnosis, including imaging like CT scan, and management of underlying etiology are necessary to manage this condition.
